# Bronchoconstriction damages airway epithelia by crowding-induced excess cell extrusion

**DOI:** 10.1126/science.adk2758

**Published:** 2024-04-04

**Authors:** Dustin C. Bagley, Tobias Russell, Elena Ortiz-Zapater, Sally Stinson, Kristina Fox, Polly F. Redd, Merry Joseph, Cassandra Deering-Rice, Christopher Reilly, Maddy Parsons, Christopher Brightling, Jody Rosenblatt

**Affiliations:** 1 The Randall Centre for Cell & Molecular Biophysics, School of Basic & Medical Biosciences, King’s College London, London SE1 1UL, UK; 2 Department of Biochemistry and Molecular Biology, University of Valencia, 46010 Valencia, Spain; 3 Institute for Lung Health, Leicester NIHR BRC, University of Leicester, Leicester LE3 9QP, UK; 4 Edwards Life Sciences, Draper, UT 84020, USA; 5 University of Utah, Salt Lake City, UT 84112, USA; 6 University of Utah School of Medicine, Salt Lake City, UT 84132, USA; 7 College of Pharmacy, University of Utah, Salt Lake City, UT 84112, USA; 8 School of Cancer and Pharmaceutical Sciences, King’s College London, London SE1 1UL, UK

## Abstract

Asthma is deemed an inflammatory disease, yet the defining diagnostic feature is mechanical bronchoconstriction. We previously discovered a conserved process called cell extrusion that drives homeostatic epithelial cell death when cells become too crowded. In this work, we show that the pathological crowding of a bronchoconstrictive attack causes so much epithelial cell extrusion that it damages the airways, resulting in inflammation and mucus secretion in both mice and humans. Although relaxing the airways with the rescue treatment albuterol did not affect these responses, inhibiting live cell extrusion signaling during bronchoconstriction prevented all these features. Our findings show that bronchoconstriction causes epithelial damage and inflammation by excess crowding-induced cell extrusion and suggest that blocking epithelial extrusion, instead of the ensuing downstream inflammation, could prevent the feed-forward asthma inflammatory cycle.

**M**ore than 300 million people globally suffer from asthma, with ~1000 dying from it daily. All asthma exacerbations are characterized by bronchoconstriction, which causes breathing difficulty, wheezing, and increased airway mucus production. After a severe exacerbation, patients with asthma frequently experience an extended period of airway inflammation for weeks to months that can predispose individuals to further attacks (1). Although diverse stimuli can trigger asthma attacks that cause different types of immune responses, the universal life-threatening pathology shared by asthmatics is mechanical bronchoconstriction. In fact, a bronchoconstrictive response to methacholine (MCH) challenge is the standard diagnostic test for asthma (2). Current therapies include albuterol, a short-acting β2 adrenergic receptor agonist that relaxes airway smooth muscle (ASM) to relieve constriction, and inhaled corticosteroids, which reduce eosinophilic and type 2 airway inflammation (3). Although these therapies help, many patients continue to experience poor symptom control and airway hyperresponsiveness. Thus, identifying other etiologies driving asthma morbidity and mortality is a priority.

Epithelia that line the airways provide a protective barrier for the lungs, acting as a first line of defense in innate immunity (4–7). Central to providing a tight barrier to the outside world is maintaining a constant density of epithelial cells while they turn over by cell division and death. We discovered a conserved process that is essential for preserving the barrier and epithelial cell number homeostasis called cell extrusion (8, 9). Extrusion mechanically links the number of cells dying with those dividing by triggering some cells to seamlessly squeeze out of the layer when it becomes too crowded (8–10). Given that mild physiological crowding causes homeostatic cell extrusion responses, we postulated that pathological crowding from a bronchoconstrictive episode might trigger so much cell extrusion that it would disrupt the airway epithelial barrier. Destruction of the airway barrier could then lead to the increased airway inflammation and/or the elevated susceptibility to viral and bacterial infection that frequently perpetuate the asthma inflammatory cycle (11). We investigated whether bronchoconstriction triggers excess airway epithelial extrusion and, if so, whether targeting extrusion could reduce the damage and inflammation, which can lead to further attacks.

## Bronchoconstriction causes excess crowding-induced extrusion

**Fig. 1:**
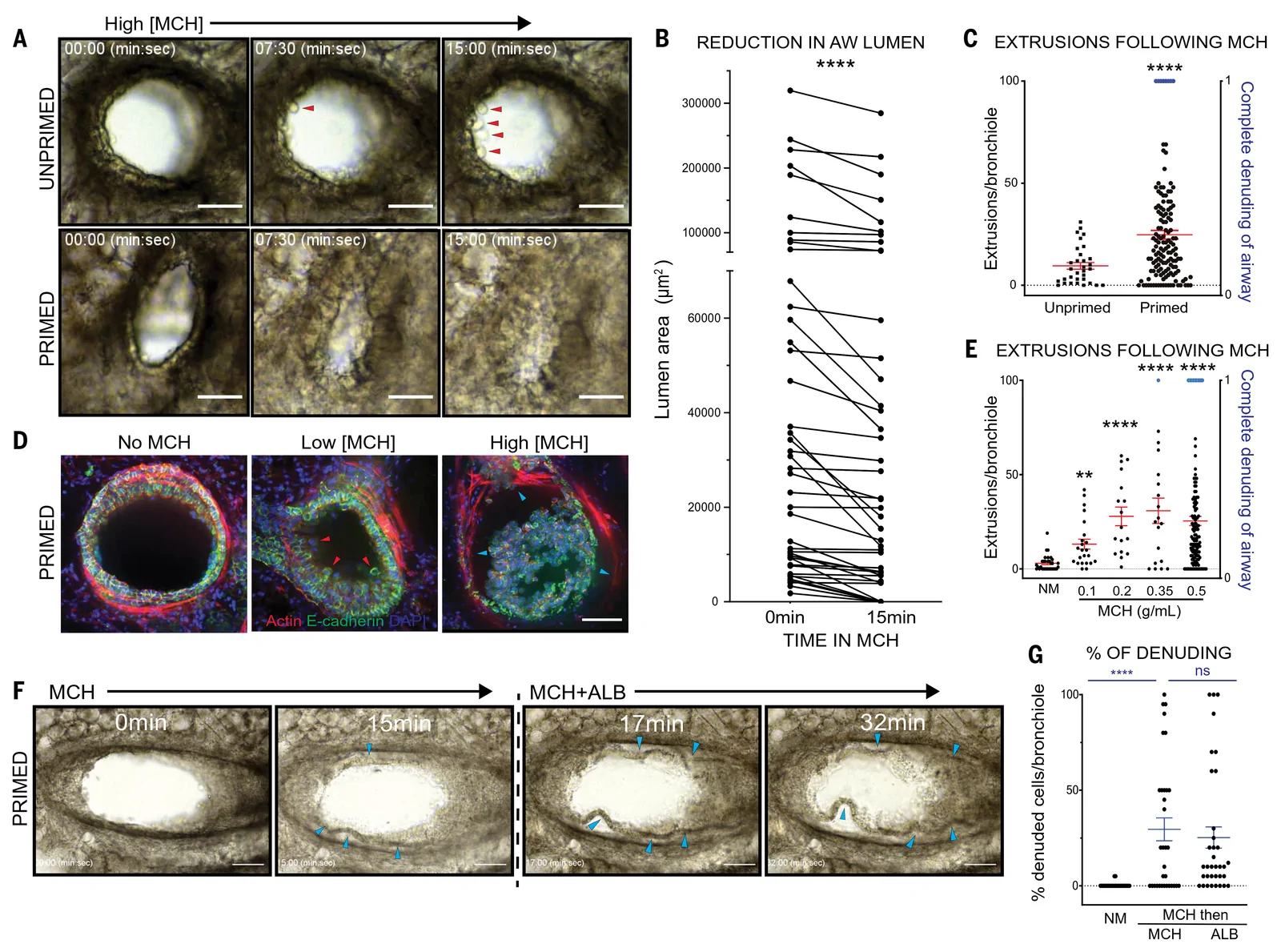
Bronchoconstriction in ex vivo lung slices causes excess cell extrusion. **(A)** Movie stills of 500 mg/ml MCH response in unprimed and 2-week HDM-primed bronchioles (minutes:seconds), with red arrowheads pointing to single-cell extrusions (scale bars, 50 µm). **(B)** Constriction scales with size, measuring the bronchiolar lumen areas before and after MCH treatment in large, medium, and small bronchioles from >5 airways from 5 HDM-primed mice (****P < 0.0001 from a Wilcoxon pairs-matched signed rank test). **(C)** Quantification of extrusions from immunostained ex vivo lung slices from unprimed and OVA-primed mice treated with 500 mg/ml MCH (****P < 0.0005 from an unpaired Mann-Whitney analysis compared with control from >5 lung slices from >10 mice), where blue data points represent complete epithelial denuding. **(D)** Sample confocal projections (scale bar, 50 µm) of bronchioles after 200 mg/ml (low) or 500 mg/ml (high) MCH, where red arrowheads depict single-cell extrusions and blue ones depict epithelial denuding (<100 extrusions on graphs; blue data points). (E) Increasing MCH concentration increases extrusion (**P < 0.0005; ****P < 0.0001) in >15 slices per treatment from >5 mice. (F and G) Movie stills (F) from a 5-week HDM-primed ex vivo lung slice treated with 500 mg/ml MCH for 15 min and then relaxed with 3.5 mM ALB + 500 mg/ml MCH for 15 min, showing that epithelia still detach (blue arrowheads; scale bars, 100 µm), quantified in (G) with no significant difference (ns) between MCH and ALB from a Mann-Whitney test (****P < 0.0001).

To test whether experimental bronchoconstriction can directly induce airway epithelial cell extrusion, we treated unprimed or immune-primed ex vivo mouse lung slices with MCH, which triggers ASM encircling the epithelial barrier to contract (12, 13). To prime the airways, we used several published ovalbumin (OVA) and house dust mite (HDM) immune-priming methods (14–19) (fig. S1, A and B) that produce characteristic asthma T helper 2 (T_H_2) cell inflammatory responses, marked by increased inflammatory cytokines interleukin-4 (IL-4) and IL-13 and high mucus production (fig. S1, C to F). MCH addition caused pronounced bronchoconstriction of mouse lungs that were immune-primed with either OVA or HDM [as outlined in fig. S1 (14–19)] but did not substantially affect unprimed airways (Fig. 1, A and B, and movies S1 to S4). Within 15 min of MCH treatment, the luminal area of small and medium bronchioles reduced markedly, causing severe airway epithelial crowding (Fig. 1B) and increasing cell heights on average 196 ± 11% (SEM; 13 airways from *n* = 5 mice). Fifteen minutes of bronchoconstriction caused excess airway epithelial extrusion in primed ex vivo mouse slices, sparing unprimed controls (Fig. 1, A and C). To quantify the number of extrusions in response to MCH, we immunostained lung slices with E-cadherin for epithelia, phalloidin for ASM, and 4′,6-diamidino-2-phenylindole (DAPI) for DNA, scoring for extrusions as cells pinching off apically into the lumen (Fig. 1, C to E, black data points and red arrowheads) and complete epithelial denuding (Fig. 1, C to E, blue data points and blue arrowheads). All immune-priming methods induced pronounced bronchoconstriction and excess extrusion in response to MCH compared with unprimed control mice, with HDM priming showing the strongest effects (Fig. 1, A and B, and movies S2 to S4). Additionally, we occasionally noted the unjamming of airway epithelia from a previous static state to collective migration (movie S5). To test whether the amount of constriction correlated with extrusion rates, we used increasing MCH doses on OVA-primed mice and found a tight correlation between the amount of constriction and cell extrusions, with the highest doses causing complete denuding of the epithelium (Fig. 1, D and E).

## Reversing bronchoconstriction does not prevent extrusion

Alleviating airway constriction with albuterol could potentially reverse the excess epithelial extrusion and denuding. However, we found that although albuterol relaxes airways that were bronchoconstricted for 15 min, it did not prevent airway epithelial extrusion and destruction (Fig. 1, F and G, and movie S6). In fact, movies show that epithelia frequently detached from the smooth muscle as the ASM sprang open with albuterol administration, whereas epithelia remained buckled (movie S6 and Fig. 1, F and G, blue arrowheads). For this reason, we quantified the percent of epithelial cell denuding per bronchiole rather than extrusions (Fig. 1G). Thus, albuterol does not prevent destruction of the airway lining after a bronchoconstrictive attack. Moreover, the increased denuding that we noted could potentially impede airway repair.

## Inhibiting extrusion blocks airway epithelial damage

**Fig. 2:**
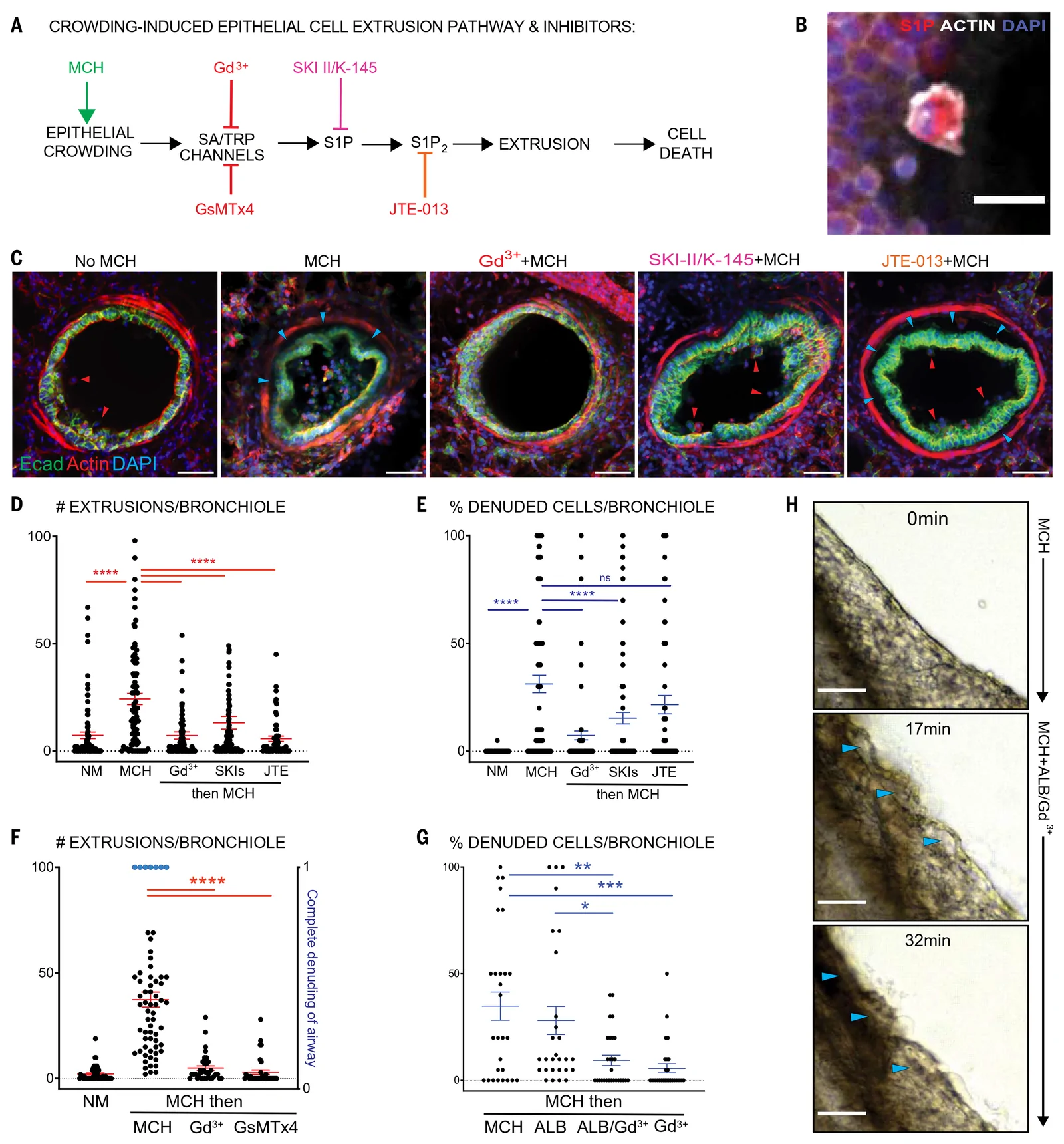
SAC and S1P inhibitors block extrusion caused by bronchoconstriction. **(A)** Canonical crowding-induced epithelial cell extrusion pathway, indicating where each inhibitor acts within the pathway. **(B)** Confocal projection of an extruding cell immunostained for S1P, phalloidin for f-actin, and DAPI (scale bar, 25 µm). (**C** to **E**) Confocal projections (C) of ex vivo lung slices from 5-week HDM-primed mice with no MCH *(n* = 9), MCH *(n* = 9), or pretreated with Gd^3+^ *(n* = 9), sphingosine kinase inhibitors SKI II and K-145 *(n* = 9), or S1P_2_ antagonist JTE-013 *(n* = 4), before adding MCH (scale bars, 50 µm), quantified in (D) as extruded cells (red arrowheads) and (E) as percentage of epithelial denuding (blue arrowheads) per bronchiole (****P > 0.0001 from a Kruskal-Wallis test, from >4 slices per mouse from >4 mice). (**F** and **G**) Quantification of OVA-primed lung slices treated with Gd^3+^ or GsMTx4 after 15 min of MCH challenge, where black dots represent extrusions per bronchiole and blue dots represent complete epithelial denuding from >5 mice (F) or percent denuding (G) (*P < 0.05; **P < 0.001; ***P < 0.0005; ****P < 0.0001 from a Mann-Whitney test, from >4 slices per mouse from >4 mice). (**H**) Movie stills from an HDM-primed mouse lung slice pretreated with MCH for 15 min, followed by ALB (3.5 mM) + MCH (500 mg/ml), with arrowheads pointing to areas of epithelial denuding that reattach by 32 min (scale bars, 50 µm). Note that albuterol alone did not prevent epithelial destruction (G).

We next investigated whether canonical extrusion inhibitors could prevent bronchoconstrictive airway damage. We previously discovered that crowding-induced live cell extrusion requires the stretch-activated channel (SAC) Piezo1 to trigger production of the bioactive lipid sphingosine 1–phosphate (S1P) that binds the S1P_2_ receptor to activate Rho-mediated actomyosin contraction needed to seamlessly eject a cell from the monolayer (8, 20) (Fig. 2A, schematic, and fig. S2A). As has been seen previously with crowding-induced extrusion (8), few of the extruding cells are apoptotic (fig. S2B). Extruding airway epithelial cells specifically up-regulate S1P, the limiting signal for extrusion, which suggests that bronchoconstrictive extrusion operates through the canonical pathway (Fig. 2B and fig. S2A). Although Piezo1—the SAC that we identified as critical for activating extrusion in response to crowding in Madin-Darby canine kidney (MDCK) cells and zebrafish (8)—is expressed in mouse airways, they also express the transient receptor protein (TRP) channels TRPA1, TRPV1, and TRPM8 frequently up-regulated in unmanageable asthma (21) that could act similarly to trigger extrusion (fig. S2, C to F). Notably, immune priming causes Piezo1, TRPV1, and TRPM8 to become more vesicular and widespread (fig. S2, C to F). Thus, to inhibit Piezo1 as well as other potential TRP channels, we used gadolinium hexahydrate chloride (Gd^3+^), an inhibitor of SACs and TRP channels. Additionally, we blocked S1P production with sphingosine kinase 1 (SKI II) and 2 (K-145) inhibitors and its receptor S1P_2_ with the antagonist JTE-013 during MCH-induced bronchoconstriction and quantified extrusion and denuding. All inhibitors markedly decreased extrusions after MCH (Fig. 2, C and D); however, only Gd^3+^ and SK inhibitors blocked epithelial sheet denuding as well (Fig. 2, C and E). It is not clear why only Gd^3+^ and SK inhibitors block detachment, but it suggests that blocking extrusion upstream in the pathway offers greater protection to airway epithelium.

We next tested whether we could inhibit extrusion in a more clinically relevant way—after the onset of bronchoconstriction and in the presence of albuterol. Thus, we triggered bronchoconstriction with MCH for 15 min and then added SAC inhibitors in the presence of MCH. Gd^3+^ administered after bronchoconstriction substantially reduced extrusion and epithelial denuding (Fig. 2, F and G). Additionally, the peptide inhibitor, GsMTx4, which blocks Piezo1, TRPC1, and TRPC6 (22), also blocks extrusion when added 15 min after bronchoconstriction (Fig. 2F). Albuterol did not impair the ability of Gd^3+^ to block epithelial extrusion or denuding after 15 min of MCH-induced bronchoconstriction, nor did Gd^3+^ affect bronchodilation by albuterol (Fig. 2, G and H, and movies S7 and S8). Our videos show that Gd^3+^ added to albuterol allows the airway epithelia to reattach to the underlying smooth muscle as it relaxes, thereby preventing denuding (Fig. 2H, blue arrowheads after they detach, with red arrowheads indicating extruded cells; movies S7 and S8). It is unclear why blocking upstream in the extrusion pathway allows epithelial reattachment, but we suggest that it prevents the secretion of matrix proteases predicted to be required during the extrusion process.

## Blocking extrusion during bronchoconstriction prevents inflammation

**Fig. 3:**
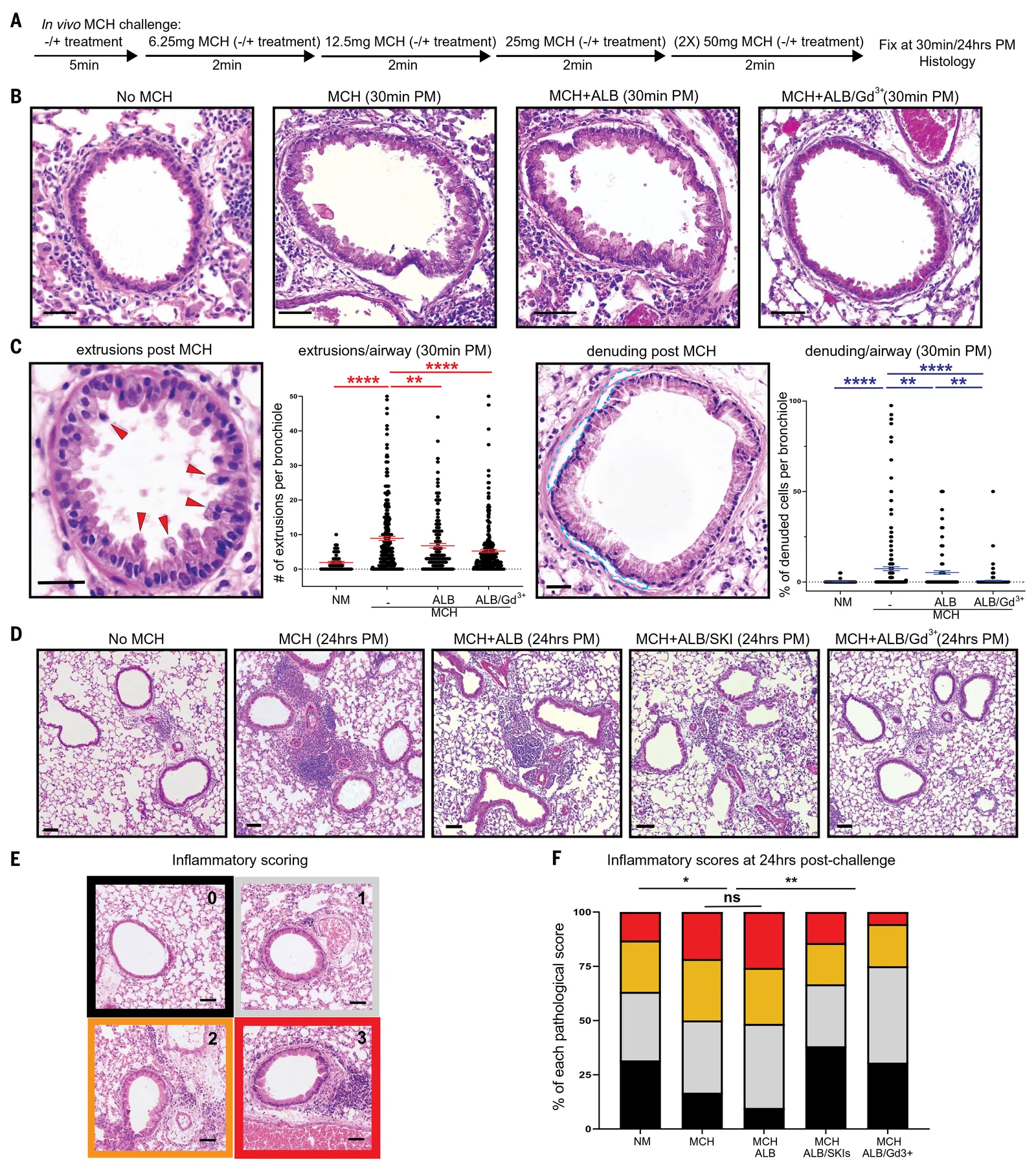
SKIs and gadolinium block extrusion and inflammation after a bronchoconstrictive attack in live mice. **(A)** Schematic of live MCH challenges ± 5 min of pretreatment with extrusion inhibitors before increasing MCH ± inhibitors. (**B** and **C**) H&E sections from lungs of mice not exposed to MCH *(n* = 4) or treated with MCH *(n* = 5), MCH + ALB *(n* = 5), or MCH + ALB/Gd^3+^ *(n* = 6) at 30 min after MCH, with the number of extrusions (red arrowheads) and percent denuding (blue dotted outline) per bronchiole quantified in (C), respectively, with representative images. Scale bars, 50 µm (**P < 0.001; ***P < 0.0005; ****P < 0.0001 from a Mann-Whitney test). (**D** to **F**) H&E sections 24 hours after MCH challenge to observe immune response from no MCH treatment *(n* = 4), MCH alone *(n* = 5), MCH + ALB *(n* = 5), MCH + ALB/SKI *(n* = 3), or MCH + ALB/Gd^3+^ *(n* = 5), with representative images shown in (D) and quantification of airway immune cell infiltration using pathological scores, defined by colors and numbers in (E) and (F) (*P < 0.05; **P < 0.001 from a Chi-squared test). Scale bars, 100 µm.

The ability of Gd^3+^ to prevent bronchoconstriction-induced airway epithelial extrusion and destruction suggested that it may prevent the inflammation that typically follows an asthma attack. To test this hypothesis, we challenged live HDM-primed mice with albuterol and inhibitors of extrusion—compared with control and MCH alone—and assayed for extrusions, denuding [30 min post-MCH (PM)], and inflammation (24 hours PM) by hematoxylin and eosin (H&E) staining. We identified 50mg/ml MCH (1/10 the concentration used in ex vivo experiments) as the lowest dose that would trigger bronchoconstriction and extrusion without causing undue harm and stress to mice, delivering it in increasing amounts, as administered with patients, to ensure that no mice were hyperresponsive (Fig. 3A, schematic). We then administered Gd^3+^ or sphingosine kinase inhibitors (SKIs) during MCH challenge to test whether blocking early steps in the extrusion pathway that protected epithelia in our ex vivo assay would prevent subsequent inflammation in our in vivo assay. SKIs can be repeatedly administered safely to mice intranasally (23), and we found that mouse lung gross morphology and tissue sections were indistinguishable from those of control mice (fig. S3A) after intranasal instillation of 10 µM Gd^3+^ for 5 consecutive days (fig. S3B) or once a week for 3 weeks (fig. S3C). We found no obvious masses, inflammation, or obvious changes to mouse health and behavior (movies S9 and S10). Because albuterol does not impede the ability of Gd^3+^ to block extrusion (Fig. 2, G and H; fig. S4C; and movies S7 and S8) and is necessary for opening airways in patients, we used albuterol with Gd^3+^ treatment in all of our live mouse studies to reduce total numbers of mice. Histology slices 30 min after MCH challenge with or without albuterol resulted in high numbers of extrusion (Fig. 3, B and C, red arrowheads) and denuded bronchioles (Fig. 3, B and C, red outline). Yet, the addition of Gd^3+^ with albuterol preserved airway epithelia, similarly to no MCH challenge (NM) (Fig. 3, B and C). The damage incurred during bronchoconstriction with or without albuterol resulted in pronounced immune cell infiltration by 24 hours compared with immune-primed unchallenged bronchioles (Fig. 3, D to F). Inhibiting extrusion with gadolinium or SKIs (with albuterol) reduced the inflammatory response to levels seen in control immune-primed, unchallenged bronchioles (Fig. 3, D to F). Similar results were obtained with OVA-primed mice, even when Gd^3+^ was delivered after the MCH ramp-up (fig. S4). Thus, preventing bronchoconstriction-induced extrusion, particularly with gadolinium, prevents the inflammation that typically follows an attack.

## SAC inhibitors also prevent mucus secretion

**Fig. 4:**
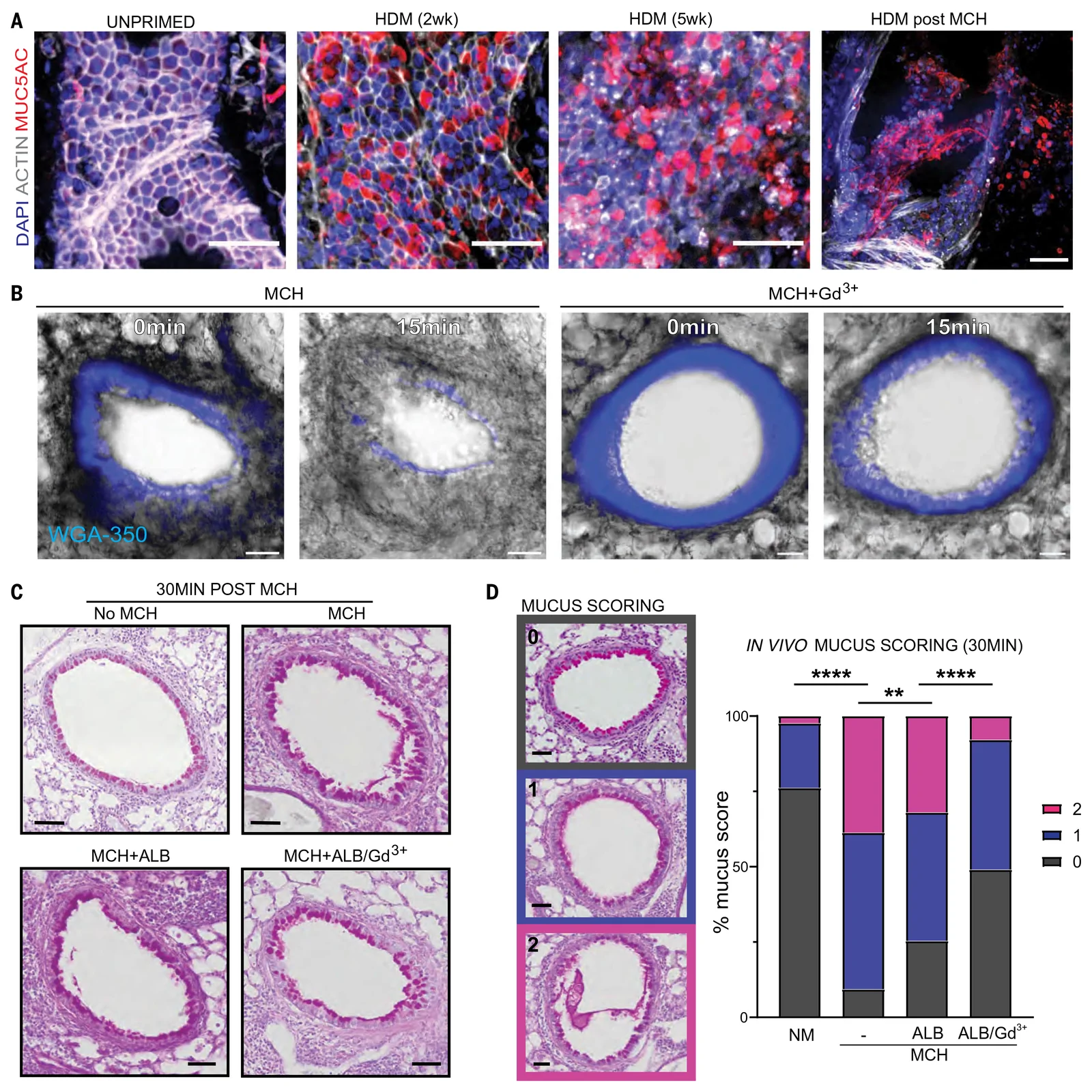
Gadolinium blocks mechanically induced mucus secretion. **(A)** Confocal projections of Muc5AC before and after 2 and 5 weeks of HDM priming (scale bars, 50 µm). **(B)** Movie stills from 3-week HDM-primed ex vivo slices incubated with 10 µg/mL WGA-350 to label mucus then treated with 500 mg/ml MCH alone ± 10 µM Gd^3+^Cl for 15 min, noting that WGA is retained in the epithelium rather than secreted with gadolinium. (**C** and **D**) Representative PAS (mucus) staining (C) 30 min after a MCH challenge ± ALB or ALB/Gd^3+^, which was quantified using a color/number scoring in (D), with **P < 0.001 and ****P < 0.0001 from Chi-square tests from at least 5 mice per group. Scale bars, 50 µm.

Although asthma typically causes greater damage to the distal airways, most asthma patients experience notable difficulties with excess mucus secretion from the proximal larger airways. Immune priming with OVA or HDM predictably increased mucus production and secretion, as measured by quantitative reverse transcription polymerase chain reaction (qRT-PCR) (fig. S1E), periodic acid–Schiff (PAS) staining of primary airways (fig. S1, E and F), and Muc5AC immunostained confocal projections (Fig. 4A) (14, 15, 24–27). Live imaging of primary airways loaded with wheat germ agglutinin–350 (WGA-350) to label mucus from 3-week HDM-primed mice revealed that MCH induces rapid mucus secretion with bronchoconstriction, as shown by the loss of blue fluorescence (Fig. 4B and movies S11 and S12). Unexpectedly, Gd^3+^ markedly reduced mucus secretion in primary airways in response to our ex vivo MCH challenges (Fig. 4B and movie S13). Although our WGA-350 assay is not a very quantitative assay because of the uneven expression of mucus from immune priming and WGA loading, we found that ~90% of control primary airways secrete mucus, whereas only ~26% of Gd^3+^-treated ones do. In vivo, we saw a similar response in both OVA- and HDM-primed mice. PAS staining from HDM-primed mice challenged with MCH for ~30 min showed bulk mucus secretion with large mucus globules squeezing out apically, which was not prevented by albuterol (Fig. 4, C and D), whereas Gd^3+^ treatment with albuterol substantially reduced mucus secretion (Fig. 4, C and D). Similar results were found in OVA-primed mice (fig. S5).

## Corticosteroid-treated asthma patients have marked airway epithelial extrusion and damage

**Fig. 5:**
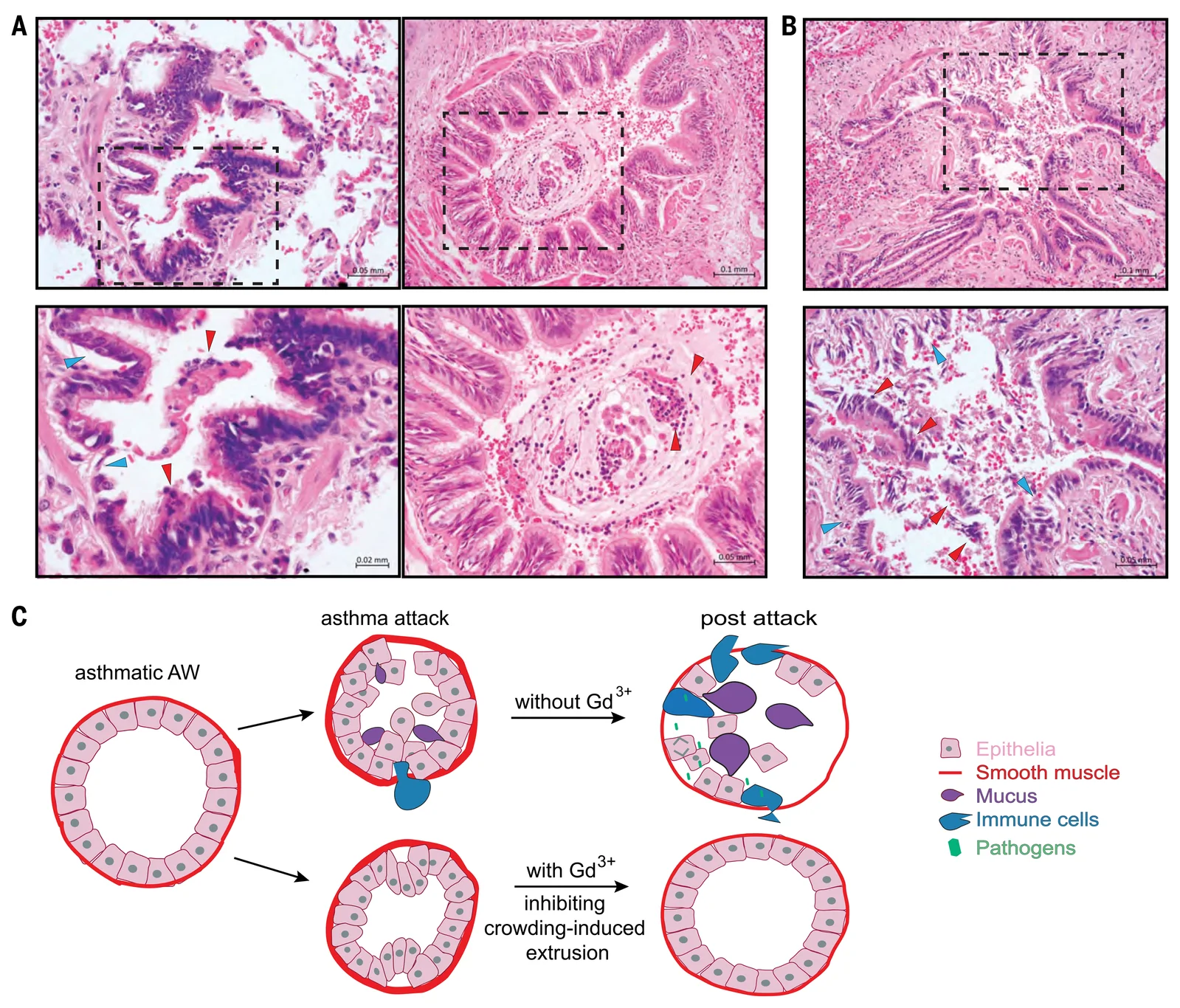
Treated patients with asthma have marked airway epithelial extrusion and damage. (**A** and **B**) H&E pathology sections from lower lobectomy of a nonsmoker patient with moderate asthma symptoms medicated with regular moderate–dose inhaled corticosteroid and long-acting β2-agonist (formoterol) and as-needed inhaled short-acting β2-agonist (albuterol) (A) and a nonsmoker patient with severe asthma on high-dose inhaled corticosteroid, long-acting β2-agonist (formoterol), oral leukotriene receptor antagonist, oral prednisone, and as-needed inhaled short-acting β2-agonist (albuterol) (B), where dashed-line boxes indicate areas enlarged below, with blue arrowheads indicating breaks in the epithelium and red arrowheads indicating epithelia extruded into the lumen. Note, lumen filled with mucus and extruded cells even in the moderate asthma case [(A), right]. (**C**) Model describing how the mechanics of asthmatic bronchoconstriction cause excessive crowding-induced mucus secretion and epithelial cell extrusion that results in epithelial damage and inflammation. Inhibiting extrusion early in the pathway prevents all pathological consequences of an attack. AW, airway.

Having found that bronchoconstriction causes excess airway epithelial cell extrusion and destruction in mice, we investigated whether a similar scenario occurs in humans. We obtained small airway resections from asthma patients undergoing lobectomy for cancer. Biopsy samples from individuals with either moderate (Fig. 5A) or severe (Fig. 5B) asthma had even more extrusion, denuding, and mucus secretion compared with our primed mice after bronchoconstriction. Both patients were treated with both corticosteroids and smooth muscle–relaxing medications, underscoring their inability to impede airway epithelial damage from asthma attacks. These individuals are not unusual, as many reports note airway epithelial sloughing or extrusion as a defining pathological feature in mild to severe or fatal cases, even in racehorses (28–30), which suggests that excess airway epithelial extrusion is a bronchoconstriction-associated pathology conserved throughout different species, independent of airway size.

## Discussion

In this work, we present a mechanical etiology for asthma. Whereas most asthma studies have focused on the inflammatory signaling associated with asthma, our work suggests that the inflammation and mucus secretion result from the mechanics of bronchoconstriction on airway epithelium. We find that bronchoconstriction causes pathological airway epithelial crowding, leading to so much cell extrusion that it destroys the barrier, resulting in the typical postattack inflammation (Fig. 5C). Because epithelia act as the first line of defense against pathogens and toxins, epithelial barrier disruption could also cause infection hypersensitivity until airways have repaired. Infections and inflammation could then lead to more bronchoconstrictive attacks, triggering the asthma inflammatory cycle (31). Albuterol treatment, routinely used by asthma patients for symptom relief of bronchospasm, does not prevent airway epithelia destruction, mucus secretion, or inflammation after an asthma attack. However, blocking the extrusion pathway with gadolinium or S1P inhibitors after bronchoconstriction preserved epithelial integrity, substantially dampening the inflammatory response. Gadolinium also blocked mucus secretion from primary airways, which suggests that crowding activation of calcium channels mechanically induces bulk mucus secretion.

Although our experiments defined excess airway epithelial cell extrusion as important for triggering many symptoms after an asthma attack, we are not the first to examine the effects of the mechanics of epithelia in asthma. Many have noted the important role of epithelia in asthma (4, 32–34). Additionally, compression forces can cause airway epithelia to unjam from their previous static, polygonal state and migrate collectively—a phenomenon linked to both asthma and idiopathic pulmonary fibrosis (35–37). We occasionally noted airway epithelial streaming or unjamming after MCH treatment, which could also contribute to the forces driving extensive extrusion and denuding of airways. The reduced cell numbers resulting from excess extrusions after strong bronchoconstriction could also trigger a wound healing response that would not only result in a poor barrier but also in matrix deposition and fibroblast activation that could promote more reactive airways.

Our studies define the extrusion pathway in controlling the downstream symptoms of an asthma attack. Admittedly, our studies do not address whether Piezo1 or TRP channels specifically control extrusion in response to bronchoconstriction. Thus, future studies will need to determine which channels respond to different asthma triggers. A variety of TRP channels are not only mutated in many cases of poorly controlled asthma but are activated by numerous stresses that trigger asthma attacks, including pollution, smoke, and cold, which suggests that several channels may be relevant (21, 38–40). Thus, Gd^3+^ has the advantage of generically blocking extrusion upstream in the extrusion pathway. Additionally, transient use of Gd^3+^ has the added benefit that it may be used practically once an attack occurs without apparent side effects in mice, but its safety will need to be tested in human airways. Gd^3+^not only serves to establish a role for mechanically induced extrusion in driving asthma symptoms but also suggests that targeting extrusion upstream in the pathway may prevent downstream symptoms. If Gd^3+^ is not safe in humans, development of other early extrusion inhibitors may provide therapeutic benefit.

Because blocking extrusion upstream in the pathway with gadolinium preserves the airway barrier after a bronchoconstrictive attack, it may also prevent the ASM remodeling associated with wound healing (41) and S1P production (23, 42, 43) that causes future attacks. Thus, preventing the mechanical damage caused by an asthma attack could pave the way for therapies that stop the whole asthma inflammatory cycle rather than treating only the downstream symptoms. Moreover, excess smooth muscle constriction may underlie other inflammatory syndromes, especially those linked with cramping, such as irritable bowel syndrome or inflammatory bowel disease, yielding therapeutic approaches for these unsolved medical problems.

## Supplementary Material

SMovie 1

SMovie 2

SMovie 3

SMovie 4

SMovie 5

SMovie 6

SMovie 7

SMovie 8

SMovie 9

SMovie 10

SMovie 11

SMovie 12

SMovie 13

Supplementary checklist

Supplementary Materials

## Data Availability

All data are available in the main text or the supplementary materials.
