# Supplementary material for: Bronchoconstriction damages airway epithelia by crowding-induced excess cell extrusion
Source: Science. Author manuscript; Available in PMC 2026 Oct 30. (PMC7619646; doi:10.1126/science.adk2758)
Supplement: Supplementary file 1 [file EMS217552-supplement-Supplementary_Materials.pdf]

Science
AAAS

Supplementary Materials for

Bronchoconstriction damages airway epithelia by crowding-induced excess cell extrusion

Dustin C. Bagley *et al*.

Corresponding author: Jody Rosenblatt, jody.rosenblatt@kcl.ac.uk

Science **384**, 66 (2024)
DOI: 10.1126/science.adk2758

The PDF file includes:

Materials and Methods
Figs. S1 to S5
References

Other Supplementary Material for this manuscript includes the following:

MDAR Reproducibility Checklist
Movies S1 to S13

Materials and Methods

Animal models and immune-priming methods

Mice at six to eight weeks of age were immunogen-sensitized in the following ways, by adapting and optimizing several protocols (Fig. S1A). OVA-priming: For intraperitoneal injections, 8 mg/mL albumin from chicken (OVA) (Sigma – A5503) was absorbed into an equal volume of Imject Alum (Thermo Fisher Scientific – 77161) before injecting 200 µl intraperitoneally (i.p.) into Balb/c mice (provided by the University of Utah), as stated in protocol in Fig. S1A. Mice were then challenged with aerosolized OVA (8 mg/mL for 15 min), as indicated in Fig. S1A for each protocol. All experiments were done within 48 h of the last OVA challenge. House Dust Mite (HDM): 6- to 8-week-old C57BL/6J mice (C57BL/6JOlaHsd from Envigo, UK) were anesthetized with isoflurane and administered 25 µg (total protein) of HDM (Citeq Biologics, Groningen, The Netherlands) in 25 µl PBS intranasally 5 times per week for 2 or 5 weeks as shown in Fig S1B. Analysis was performed 24 hours after the final challenge. Mice numbers are listed in figure legends of appropriate experiments and were determined in accordance with the 3Rs principles. We used mice primed as in (OVA A) for Figs. 1C-D&E, 2F&G, as in (OVA B) for Supplemental Figs. 4&5 and Supplemental Movie 3, as in (HDM A) for Figs. 1A&F, 2C-E, 3A, and Supplemental Movies 2&4-6, and as in (HDM B) for Figs. 1B, 3, 4A-D, Supplemental Fig. 2, and Supplemental Movies 3, 9-11.

All animals were maintained under specific pathogen–free conditions and handled in accordance with the Institutional Committees on Animal Welfare of the UK Home Office Animals (Scientific Procedures) Act 1986. All animal experiments were approved by the Ethical Review Process Committee at King's College London and carried out under license from the Home Office, UK. All mice were humanely handled, according to an IACUC license 16-08007 from the University of Utah and a Program Project License P68983265 to Jody Rosenblatt and P9672569A to Maddy Parsons at King's College London.

Precision-cut ex vivo lung slices (PCLSs)

Ex vivo lung slices were obtained from mice, within 48 h of their last allergen priming challenge, adapted from the protocol of (44). Briefly, mice were humanely euthanized by CO2 inhalation followed by cervical dislocation or exsanguination via the femoral artery. The chest cavity was opened and the trachea carefully exposed, where a small incision was made to accommodate the insertion of a 20Gx1.25 in canula (SURFLO I.V. catheter). The lungs were inflated with 2% low melting agarose (Fisher – BP1360) prepared in HBSS+ (Gibco – 14025) before lungs, along with the heart and trachea, were excised, washed in PBS, and the lobes separated. Individual lobes were then embedded in 4% low melting agarose and solidified on ice. 200-micron thick slices were cut on a Leica VT1200S vibratome and washed and incubated in DMEM/F-12 medium supplemented with 10% fetal bovine serum (FBS) and antibiotics. The ex vivo lung slices remained viable (MCH-reactive) for at least one week after isolation (45)

Methacholine (MCH) treatment and live imaging

Ex vivo lung slices were experimentally treated with increasing dosages of MCH (acetyl-β-methylcholine chloride, Sigma A2251), from 100 mg/mL to 500 mg/mL in HBSS+ solution. MCH is a non-selective muscarinic receptor agonist clinically used to test the severity of asthma in patients. Lungs were incubated in HBSS+ in 24-well plates at 37°C with MCH for 15-30 minutes and filmed at 15-30 sec intervals using a Life Technologies EVOS FL Auto microscope to measure bronchoconstriction in response to MCH. For lumen reduction measurements in

response to MCH, the lumen area (the "dead" or empty space in the airway lumen) at time=0 min and at time=15 min of MCH treatment was measured using Fiji software from live imaging stills. In the wheat germ agglutinin (WGA) experiments, ex vivo lung slices were pre-incubated with 10 µg/mL of WGA AlexaFluor350 (Invitrogen W11263) per manufacturer's instructions at 37°C before MCH treatment and live imaging as above. In some experiments, 10 µM gadolinium (III) chloride hexahydrate (Sigma G7532) and/or 3.5 mM albuterol (Sigma PHR1053), was used either before or 15 minutes into a MCH challenge. The SIP inhibitors at 30 µM SKI II (Sigma S5696) and K-145 (Merck 5091060001), or JTE-013 (Sigma J4080) were added at the beginning of a MCH challenge. The ex vivo lung slices were fixed in 4% paraformaldehyde overnight at 4°C before immunostaining.

Immunofluorescence and imaging of fixed PCLS

PFA-fixed ex vivo lung slices were incubated for one hour at room temperature in blocking solution: PBS containing 0.1% triton X-100, 0.1% sodium azide, and 2% bovine albumin (BSA), before incubating overnight at 4°C at 1:100 in blocking solution for all primary antibodies used: rabbit anti-E-Cadherin (24E10 – Cell Signaling 3195), rabbit anti-Piezo1 (Novus NBP-78537), mouse anti-S1P (Echelon Biosciences ZP300), mouse anti-Muc5AC (Abcam ab3649), rabbit anti-TRPV1 (Alomone ACC-030),rabbit anti-TRPV4 (Alomone ACC-034), rabbit anti-TRPA1 (Alomone ACC-037), rabbit anti-TRPM4 (Alomone ACC-044), and rabbit anti-TRPM8 (Alomone ACC-049). Ex vivo lung slices were washed 3 X 30 minutes in PBS+0.5% Triton X-100) before incubating overnight at 4°C overnight with: 1:100 Alexa Fluor 488 goat anti-rabbit (Thermo Scientific - A11008) or anti-mouse (A32723) IgG, Alexa Fluor 568 goat anti-rabbit (A11011) or anti-mouse (A11004) IgG, or Alexa Fluor 647 goat anti-rabbit (A32733) or anti-mouse (A21235) IgG + 1:250 Alexa Fluor 488, 568, or 647 Phalloidin (Thermo Scientific – A12379, A12380, A22287, respectively). Slices were washed 2 X 30 minutes in PBS+0.5% Triton X-100, stained with 1:1000 DAPI in PBS for 20 minutes, mounted in ProLong Gold (Invitrogen P36930), and imaged on a Nikon Eclipse Ti2 spinning disc confocal microscope with a 20X or 40X objective.

MCH challenge in live mice with and without treatments

Mice at the end of their immune-priming protocol were placed in a 6.5-quart Hefty bin fitted with an Aerogen Pro Nebuliser System (AG-AP6000-XX) that produces 2.5-4.0 µm volume mean diameter aerosolized particles, adapted from (46). Mice were nebulized with two-minute challenges of increasing concentrations of MCH (6.25, 12.5, 25, then 2 X 50 mg/mL), resting in fresh air for 2 minutes between challenges, with albuterol (3.5 mM) and/or gadolinium (10 µM), and/or SKIs (30 µM) given in the last three challenges, with the highest MCH concentration of 50 mg/mL. Mice were humanely euthanized at 30 min or 24 h post MCH challenge (-/+ treatments).

Histological analysis and quantification

At the end of their treatments, mice were humanely euthanized by CO2 inhalation followed by cervical dislocation or exsanguination via the femoral artery. The lungs were inflated with 10% neutral buffered formalin (NBF), excised, and fixed in NBF overnight at room temperature, followed by another overnight room temperature incubation in 70% ethanol. The large left lung, and the cranial, medial, and caudal lobes of the right lung were excised and embedded in paraffin. Using a keratome, 3X5 µm thick serial slices were made per slide, 3 slides per lung,

each slice made at 20-50 μm deeper intervals, and stained with hematoxylin and eosin (H&E) or periodic acid-Schiff (PAS). Histological grades depicted in Fig. 3 and Fig. S4 were used to determine immune cell infiltrate (H&E) and mucus phenotypes (PAS) in Fig. 4 and Fig. S5, with at least 30 airways spanning multiple lung lobes from at least five mice analyzed for each group. H&E stained lung slices were also used to determine the number of extrusions and percent denuding per bronchiole, with five mice analyzed per group (Fig. 3C&D).

Human tissue pathology

Tissue samples were supplied by The Leicester Institute for Lung Health Research Tissue Bank with the support of the NIHR Leicester Biomedical Research Centre Respiratory Theme.

Quantitative real-time PCR analysis

To confirm the induction of a Th2 inflammatory response from our immune priming protocols, the transcript levels of IL-4, IL-13, and Muc5AC (Thermo Fisher TaqMan probes – cat. # 4331182, 4331182, and 4331182, respectively) were measured by quantitative real-time PCR from cDNA transcribed (Thermo Fisher SuperScript cat. # - 18091050) from total RNA isolated from whole lung extracts (Qiagen – RNeasy Plus – cat. # 74034) and mRNA expression analyzed by a comparative Ct method.

Statistical analysis

To compare sample means, we used a non-parametric Mann–Whitney U test (47). For airway lumen constriction measurements in response to MCH treatment, a Wilcoxon pairs-matched signed rant test was used. To compare more than two groups, we used a Mann–Whitney U test, with the Kruskal-Wallis test or the Holm-Sidak adjustment for pairwise comparisons. For categorical data we used the Chi-Square test to reject the null hypothesis with the Yates correction when analyzing two populations and two categories. For analysis of categorical data, when at least 20% of the groups presented frequencies lower than 5 for a given variable, we used the Fisher Exact test.

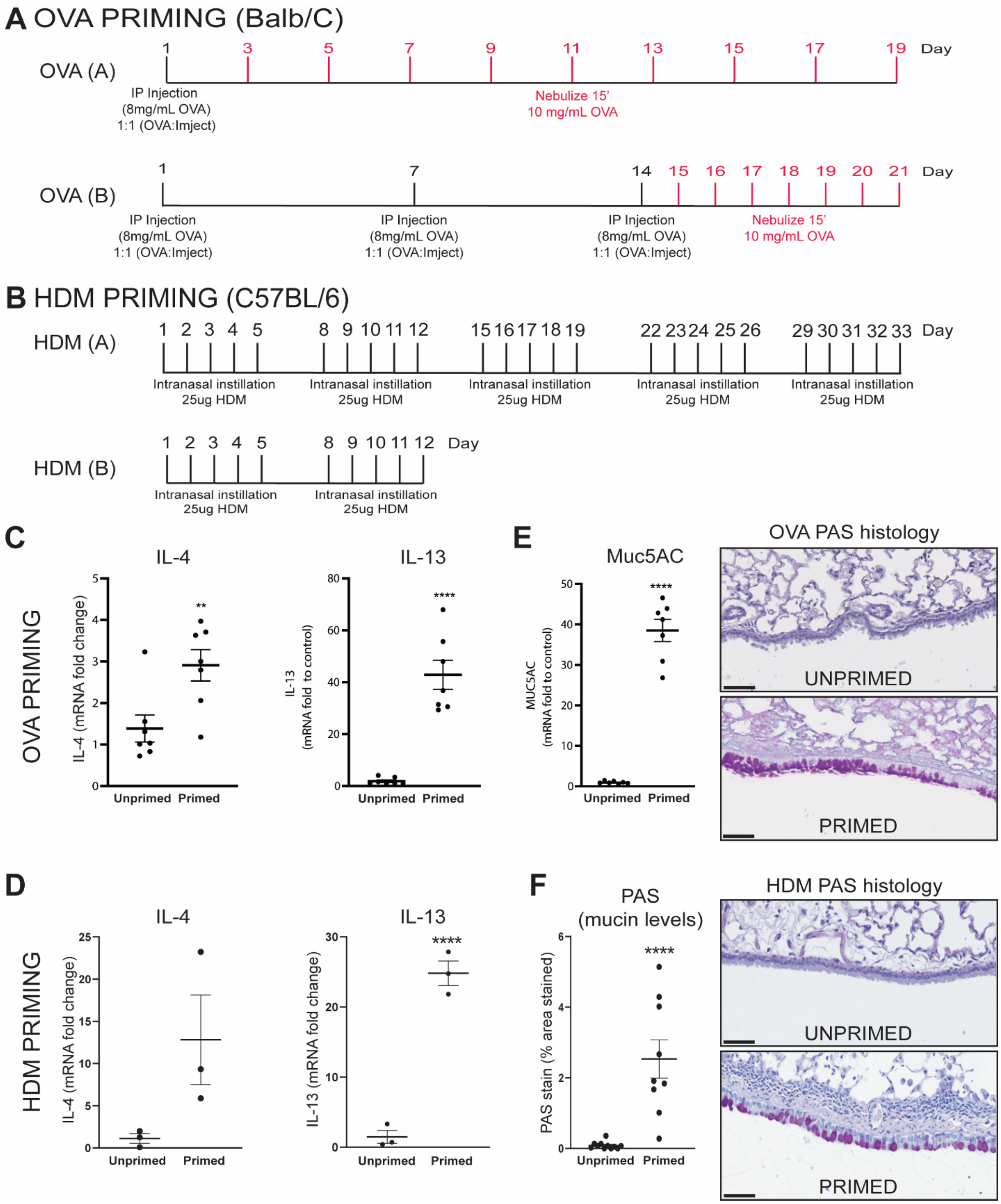

A OVA PRIMING (Balb/C)
OVA (A)
1 3 5 7 9 11 13 15 17 19 Day
IP Injection
(8mg/mL OVA)
1:1 (OVA:Imject)
Nebulize 15'
10 mg/mL OVA
OVA (B)
1 7 14 15 16 17 18 19 20 21 Day
IP Injection
(8mg/mL OVA)
1:1 (OVA:Imject)
Nebulize 15'
10 mg/mL OVA
B HDM PRIMING (C57BL/6)
HDM (A)
1 2 3 4 5 8 9 10 11 12 15 16 17 18 19 22 23 24 25 26 29 30 31 32 33 Day
Intranasal instillation
25ug HDM
HDM (B)
1 2 3 4 5 8 9 10 11 12 Day
Intranasal instillation
25ug HDM
C
OVA PRIMING
IL-4
IL-4 (mRNA fold change)
Unprimed Primed
**
IL-13
IL-13 (mRNA fold to control)
****
E
Muc5AC
MUC5AC (mRNA fold to control)
****
OVA PAS histology
UNPRIMED
PRIMED
D
HDM PRIMING
IL-4
IL-4 (mRNA fold change)
IL-13
IL-13 (mRNA fold change)
****
F
PAS (mucin levels)
PAS stain (% area stained)
****
HDM PAS histology
UNPRIMED
PRIMED

Fig. S1. Allergen-induced asthmatic mouse models

Allergen-induced asthmatic mouse models Schematics of two different immune priming protocols each for OVA-priming using Balb/c mice (A), where OVA(A) is shorter but more frequent and OVA(B) is longer with more injections with imject first and for HDM-priming using C57BL/6J mice (B, where HDM(A) is 5 weeks nasal installation every weekday and HDM(B) is 2 weeks every weekday. All priming protocols produced a characteristic Th2 inflammatory response where IL-4- and IL-13 mRNA was analyzed by qRT-PCR from OVA B (C) and HDM A (D) primed mice, with at least 3 mice per group and P**<0.005, P****<0.0001. Immune priming resulted in excessive mucus production with increased mRNA levels of the pathological mucin Muc5AC and increase PAS staining from histological sections in OVA(A) (E) and HDM(A) (F) primed mice. Scale bar = 50μm.

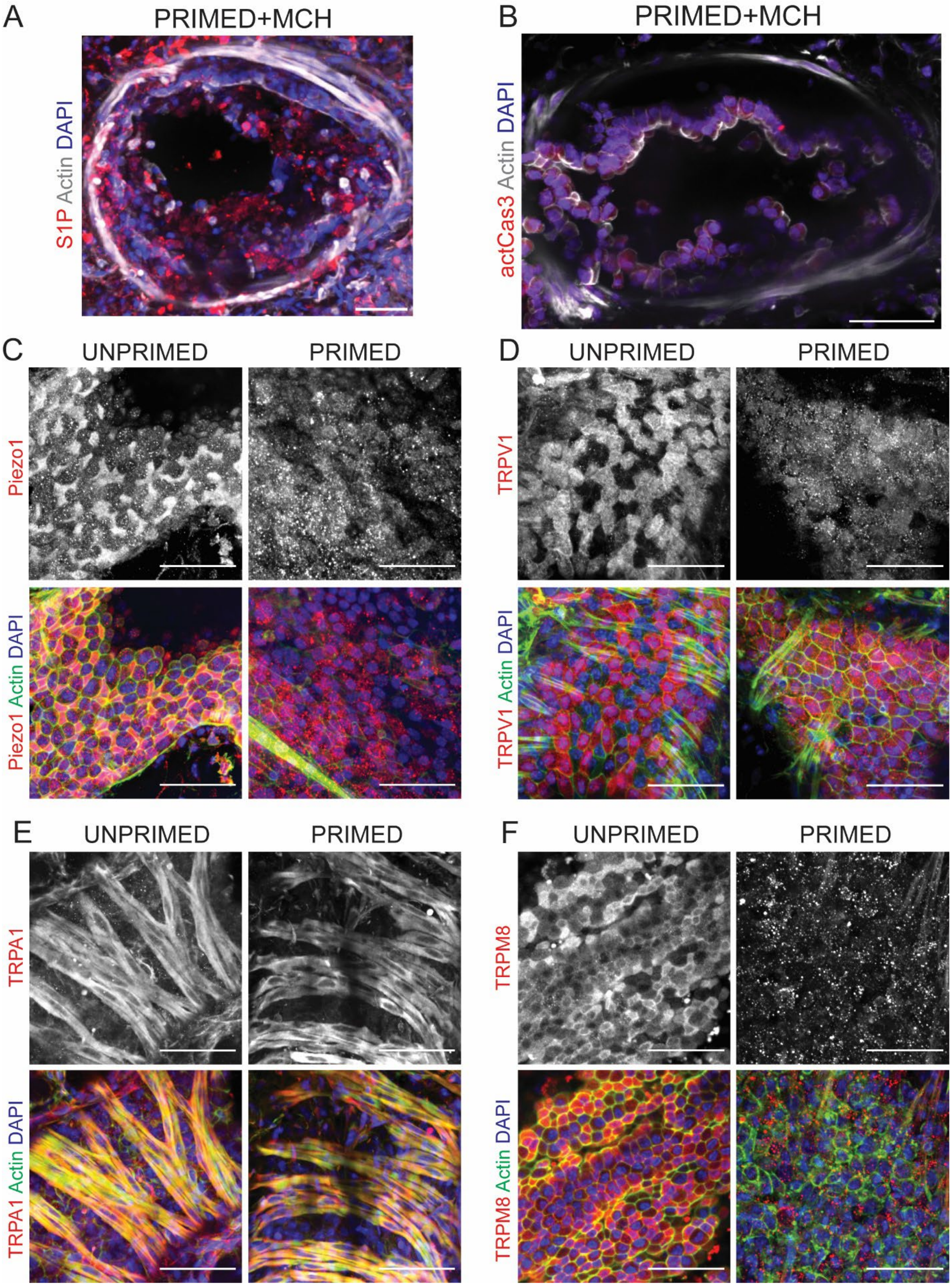
A
PRIMED+MCH
S1P Actin DAPI
B
PRIMED+MCH
actCas3 Actin DAPI
C
UNPRIMED
PRIMED
Piezo1
Piezo1 Actin DAPI
D
UNPRIMED
PRIMED
TRPV1
TRPV1 Actin DAPI
E
UNPRIMED
PRIMED
TRPA1
TRPA1 Actin DAPI
F
UNPRIMED
PRIMED
TRPM8
TRPM8 Actin DAPI

Fig. S2. Stretch-activated and Transient Receptor Potential channels in airway epithelia

Stretch-activated and Transient Receptor Potential channels in airway epithelia Confocal projections of HDM primed airways 15 min after 0.5 g/mL MCH treatment stained for S1P (A) or active caspase 3 (B), actin, and DAPI. Representative confocal projections of airways from unprimed (Ctrl) and HDM-primed mice of channels that can be inhibited by gadolinium treatment: Piezo1 (C), TRPV1 (D), TRPA1 (E), and TRPM8 (F) from >3 mice per group. (scale bars=50 μm).

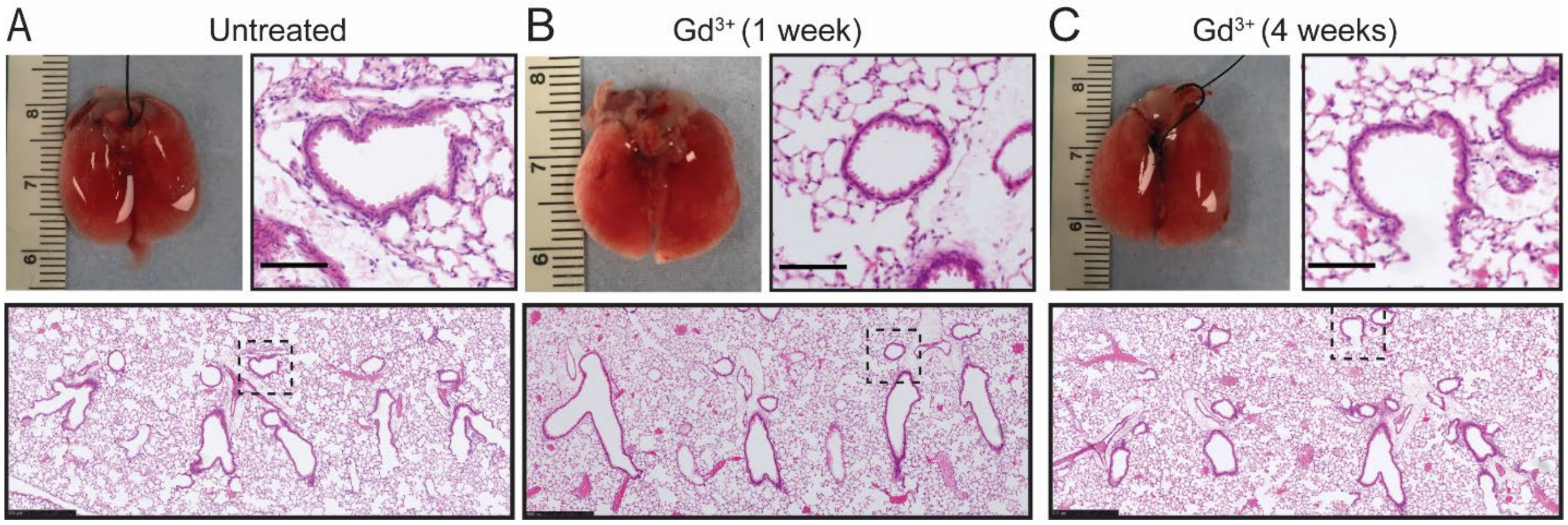

Fig. S3. Gadolinium treatment does not harm mice

Representative pictures of control mouse lungs and tissues (A) or those from mice with intranasal instillation of 10 µM Gd3+ for 5 consecutive days (B) or once per week for 4 weeks (C). No discernable differences were noted between the groups, analyzing gross lung morphology and lung pathology by H&E staining (inset scale bar=100 µm, whole image scale bar=500 µm) from 3 lung slices per mouse and 4 mice per group.

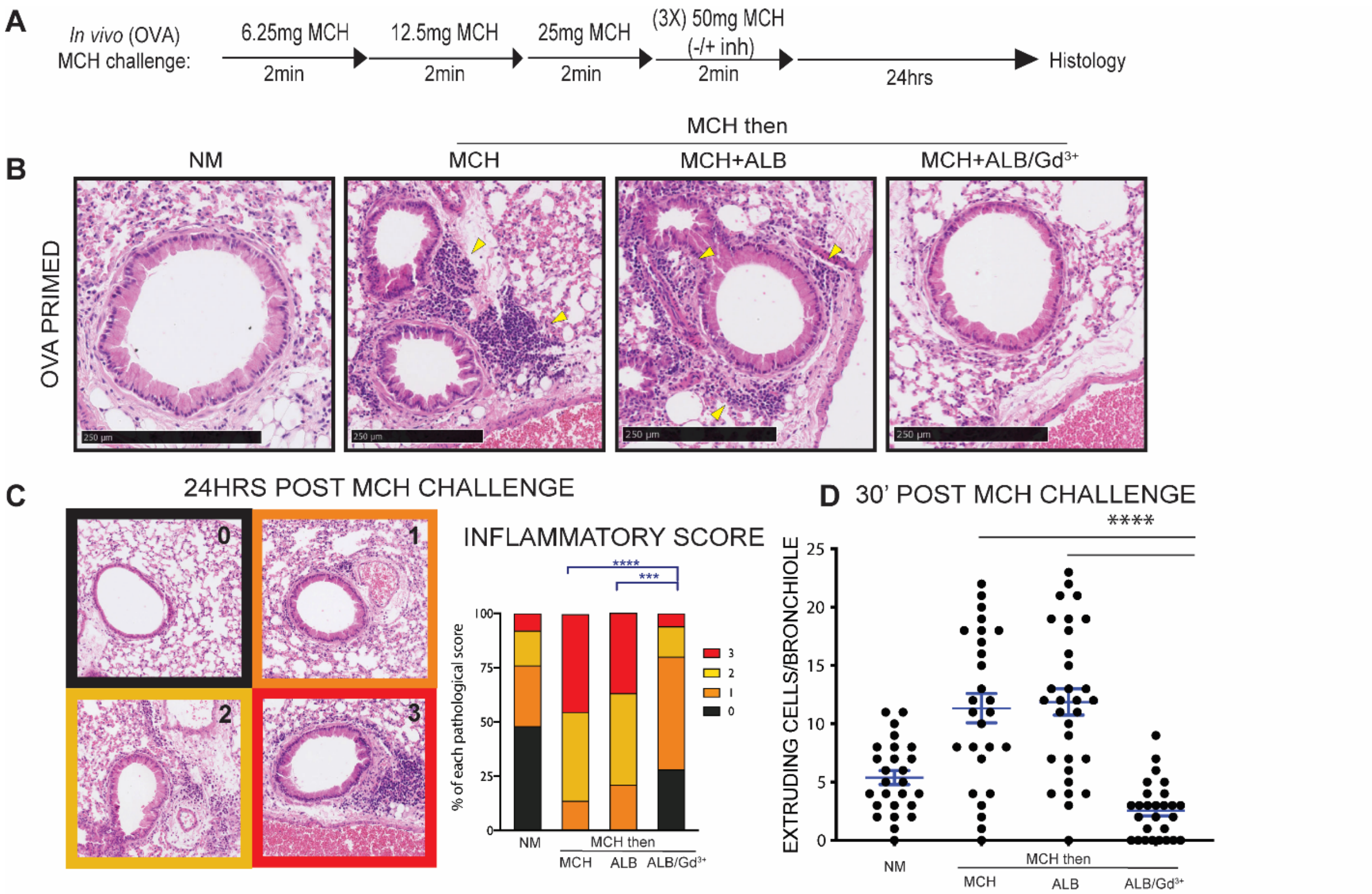

Fig. S4. Gadolinium prevents extrusion and inflammation in OVA-primed mice

(A) Schematic of in vivo MCH challenges ± ALB or ALB/Gd3+ in OVA primed mice, fixing for histology 24 hours post-challenge. (B) Representative H&E sections from lungs fixed at 24 h after live mice were treated with: no MCH (NM) or with MCH ramp for 15 min, then MCH, MCH+ALB, or MCH+ALB/Gd3+, with yellow arrowheads indicating regions of high inflammatory cell infiltrates (scale bar=250 µm), quantified in (C) for 5 mice from each treatment, with P***<0.0005 and P****<0.0001 from Chi-square tests. (D) quantification of extrusion per bronchiole at 30 min PM with P from Mann-Whitney Test

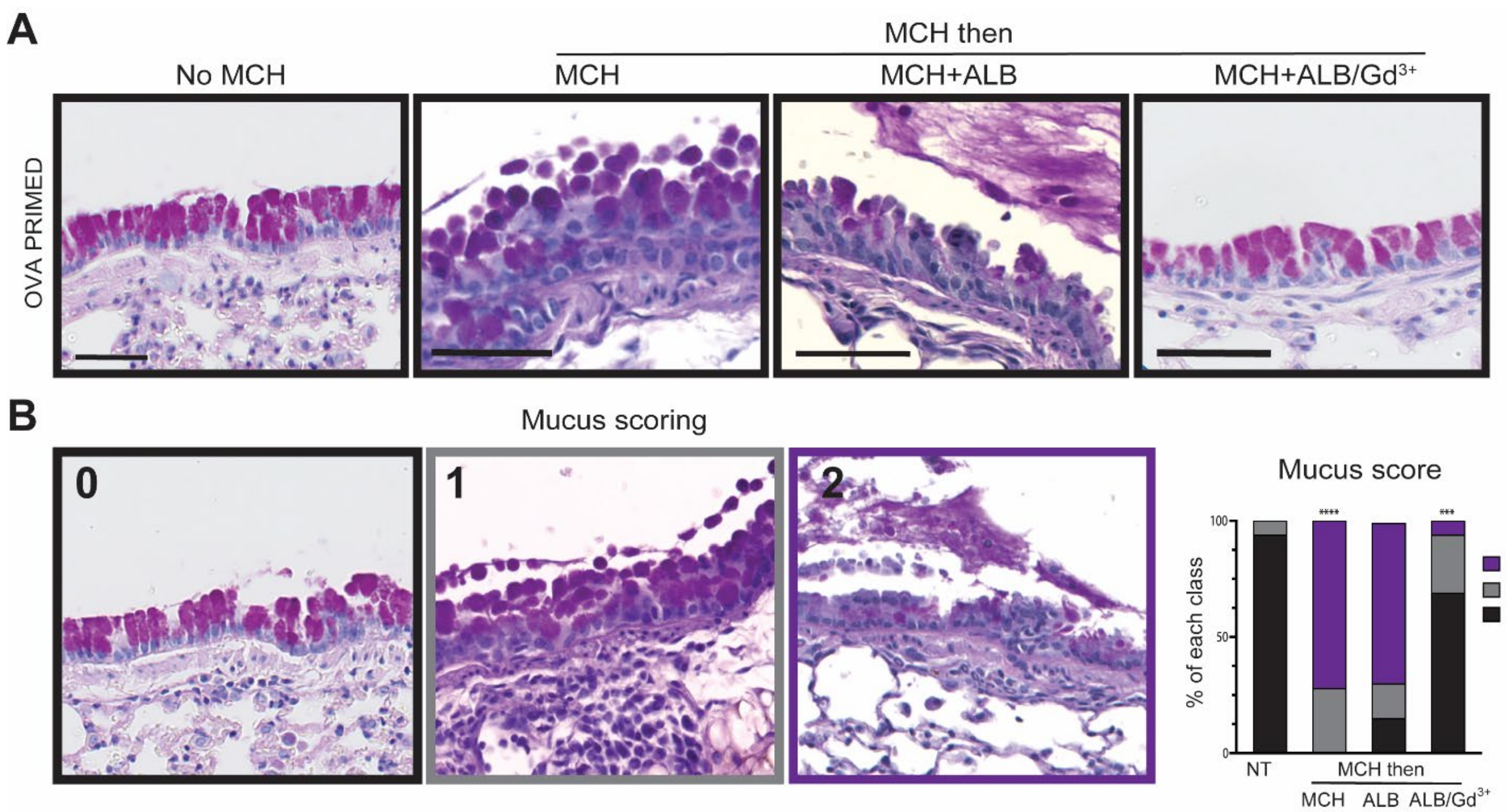

Fig. S5. Gadolinium prevents MCH-induced mucus secretion in live OVA-primed mice

(A) Representative PAS stained sections from OVA-primed mice that were not treated or treated with MCH for 15 min before treating with MCH, MCH+ALB, or MCH+ALB/Gd3+, quantified (as in Figure 4D) with a color/number score in (B). From 5 mice per treatment group with P***<0.0005 and P****<0.0001 from Chi-square tests

Movie S1. Unprimed bronchioles mildly respond to MCH treatment.
Ex vivo lung slice from a healthy control mouse treated with 0.5 g/mL MCH and imaged live by brightfield for 15 minutes, with arrowheads marking extruding cells (Fig. 1A).

Movie S2. HDM priming airways results in pathological crowding and extrusion.
Lung slice from HDM B primed mouse was imaged live for 15 min of a 0.5 g/mL MCH challenge (Fig. 1A).

Movie S3. OVA priming airways results in pathological crowding and extrusion.
Lung slice from OVA A primed mouse was treated with 0.5 g/mL MCH and imaged live for 30 min.

Movie S4. Epithelial extrusions seen in larger HDM primed airways treated with MCH.
Large airway from a HDM primed mouse treated with 0.5 g/mL MCH for 15 min (red arrowheads highlighting epithelial cells extruding above the monolayer).

Movie S5. Unjamming of epithelia following bronchoconstriction.
Large airway from a HDM primed mouse treated with 0.5 g/mL MCH for 15 min, where epithelial cells in the top bronchiole move together in a sheet, an occasional occurrence.

Movie S6. Albuterol does not reverse epithelial cell extrusion during an attack.
A medium size airway from a five-week HDM-primed mouse treated with MCH for 15 min, then MCH with albuterol (ALB) for an additional 15 min (Fig. 1F). Red arrowheads highlight sites where epithelial sheet detaches from the underlying airway smooth muscle.

Movie S7. Gadolinium protects airway epithelium even after an attack begins.
Airways from a five-week HDM-primed mouse treated with MCH for 15 min and then with MCH+ALB+Gd3+ for another 15 min. Red arrowheads pointing to areas of epithelial detachment, then Gd3+-induced reattachment by the end of the treatment, shown with blue arrowheads (Fig. 2H).

Movie S8. Gadolinium protects airway epithelium even after an attack begins.
The uncropped Supplemental Movie 7 & Fig. 2H.

Movie S9. Gadolinium does not appear to negatively impact mouse behavior.
Mice acutely treated with gadolinium on day 1 demonstrate normal grooming and social behavior.

Movie S10. Gadolinium does not appear to negatively impact mouse behavior.

Mice acutely treated with gadolinium for 5 consecutive days demonstrate normal grooming and social behavior.

Movie S11. Mechanically-induced mucus secretion in ex vivo slices by live WGA-350 imaging.

Inset of a bronchiole from a three-week HDM-primed lung pre-treated with 10 µg/mL WGA before imaging for 15 min with 0.5 g/mL MCH, showing mucus (as indicated by blue WGA) secreted from the epithelium in response to bronchoconstriction.

Movie S12. Mechanically-induced mucus secretion in ex vivo slices by live WGA-350 imaging.

A bronchiole from a three-week HDM-primed lung was pre-treated with 10 µg/mL WGA before imaging for 15 min with 0.5 g/mL MCH, demonstrating mucus (blue WGA) secreted from the epithelium in response to bronchoconstriction.

Movie S13. Gadolinium pretreatment prevents mechanically induced mucus secretion.

Ex vivo lung slices from three-week HDM-primed mice were pretreated with WGA and Gd^{3+} (10 min) then given 0.5 g/mL MCH (t=00:00) and imaged live for 15 min, demonstrating that most mucus is retained in the epithelium during bronchoconstriction.

References and Notes

1. P. G. Holt, P. D. Sly, Viral infections and atopy in asthma pathogenesis: New rationales for asthma prevention and treatment. *Nat. Med.* **18**, 726–735 (2012). doi:10.1038/nm.2768 Medline

2. R. J. Hopp, A. K. Bewtra, N. M. Nair, R. G. Townley, Specificity and sensitivity of methacholine inhalation challenge in normal and asthmatic children. *J. Allergy Clin. Immunol.* **74**, 154–158 (1984). doi:10.1016/0091-6749(84)90279-3 Medline

3. F. Menzella, C. Galeone, G. Ghidoni, P. Ruggiero, M. D'Amato, M. Fontana, N. Facciolongo, The pharmacoeconomics of the state-of-the-art drug treatments for asthma: A systematic review. *Multidiscip. Respir. Med.* **16**, 787 (2021). doi:10.4081/mrm.2021.787 Medline

4. S. T. Holgate, Epithelium dysfunction in asthma. *J. Allergy Clin. Immunol.* **120**, 1233–1244 (2007). doi:10.1016/j.jaci.2007.10.025 Medline

5. S. T. Holgate, Novel targets of therapy in asthma. *Curr. Opin. Pulm. Med.* **15**, 63–71 (2009). doi:10.1097/MCP.0b013e32831da867 Medline

6. C. G. Persson, Epithelial cells: Barrier functions and shedding-restitution mechanisms. *Am. J. Respir. Crit. Care Med.* **153**, S9–S10 (1996). doi:10.1164/ajrccm/153.6_Pt_2.S9 Medline

7. C. Xiao, S. M. Puddicombe, S. Field, J. Haywood, V. Broughton-Head, I. Puxeddu, H. M. Haitchi, E. Vernon-Wilson, D. Sammut, N. Bedke, C. Cremin, J. Sones, R. Djukanović, P. H. Howarth, J. E. Collins, S. T. Holgate, P. Monk, D. E. Davies, Defective epithelial barrier function in asthma. *J. Allergy Clin. Immunol.* **128**, 549–556.e12 (2011). doi:10.1016/j.jaci.2011.05.038 Medline

8. G. T. Eisenhoffer, P. D. Loftus, M. Yoshigi, H. Otsuna, C.-B. Chien, P. A. Morcos, J. Rosenblatt, Crowding induces live cell extrusion to maintain homeostatic cell numbers in epithelia. *Nature* **484**, 546–549 (2012). doi:10.1038/nature10999 Medline

9. J. Rosenblatt, M. C. Raff, L. P. Cramer, An epithelial cell destined for apoptosis signals its neighbors to extrude it by an actin- and myosin-dependent mechanism. *Curr. Biol.* **11**, 1847–1857 (2001). doi:10.1016/S0960-9822(01)00587-5 Medline

10. G. T. Eisenhoffer, J. Rosenblatt, Bringing balance by force: Live cell extrusion controls epithelial cell numbers. *Trends Cell Biol.* **23**, 185–192 (2013). doi:10.1016/j.tcb.2012.11.006 Medline

11. J. R. Castillo, S. P. Peters, W. W. Busse, Asthma Exacerbations: Pathogenesis, Prevention and Treatment. *J. Allergy Clin. Immunol. Pract.* **5**, 918–927 (2017). doi:10.1016/j.jaip.2017.05.001 Medline

12. M. M. Gueders, G. Paulissen, C. Crahay, F. Quesada-Calvo, J. Hacha, C. Van Hove, K. Tournoy, R. Louis, J.-M. Foidart, A. Noël, D. D. Cataldo, Mouse models of asthma: A comparison between C57BL/6 and BALB/c strains regarding bronchial responsiveness, inflammation, and cytokine production. *Inflamm. Res.* **58**, 845–854 (2009). doi:10.1007/s00011-009-0054-2 Medline

13. S. R. Rosner, S. Ram-Mohan, J. R. Paez-Cortez, T. L. Lavoie, M. L. Dowell, L. Yuan, X. Ai, A. Fine, W. C. Aird, J. Solway, J. J. Fredberg, R. Krishnan, Airway contractility in the precision-cut lung slice after cryopreservation. *Am. J. Respir. Cell Mol. Biol.* **50**, 876–881 (2014). doi:10.1165/rcmb.2013-0166MA Medline

14. W. R. Henderson Jr., D. B. Lewis, R. K. Albert, Y. Zhang, W. J. Lamm, G. K. Chiang, F. Jones, P. Eriksen, Y. T. Tien, M. Jonas, E. Y. Chi, The importance of leukotrienes in airway inflammation in a mouse model of asthma. *J. Exp. Med.* **184**, 1483–1494 (1996). doi:10.1084/jem.184.4.1483 Medline

15. W. R. Henderson Jr., L.-O. Tang, S.-J. Chu, S.-M. Tsao, G. K. S. Chiang, F. Jones, M. Jonas, C. Pae, H. Wang, E. Y. Chi, A role for cysteinyl leukotrienes in airway remodeling in a mouse asthma model. *Am. J. Respir. Crit. Care Med.* **165**, 108–116 (2002). doi:10.1164/ajrccm.165.1.2105051 Medline

16. J. R. Johnson, R. E. Wiley, R. Fattouh, F. K. Swirski, B. U. Gajewska, A. J. Coyle, J.-C. Gutierrez-Ramos, R. Ellis, M. D. Inman, M. Jordana, Continuous exposure to house dust mite elicits chronic airway inflammation and structural remodeling. *Am. J. Respir. Crit. Care Med.* **169**, 378–385 (2004). doi:10.1164/rccm.200308-1094OC Medline

17. A. Tomkinson, C. Duez, G. Cieslewicz, J. C. Pratt, A. Joetham, M.-C. Shanafelt, R. Gundel, E. W. Gelfand, A murine IL-4 receptor antagonist that inhibits IL-4- and IL-13-induced responses prevents antigen-induced airway eosinophilia and airway hyperresponsiveness. *J. Immunol.* **166**, 5792–5800 (2001). doi:10.4049/jimmunol.166.9.5792 Medline

18. K. G. Tournoy, J. C. Kips, C. Schou, R. A. Pauwels, Airway eosinophilia is not a requirement for allergen-induced airway hyperresponsiveness. *Clin. Exp. Allergy* **30**, 79–85 (2000). doi:10.1046/j.1365-2222.2000.00772.x Medline

19. K. Ulrich, J. S. Hincks, R. Walsh, E. M. C. Wetterstrand, M. D. Fidock, S. Sreckovic, D. J. Lamb, G. J. Douglas, M. Yeadon, C. Perros-Huguet, S. M. Evans, Anti-inflammatory modulation of chronic airway inflammation in the murine house dust mite model. *Pulm. Pharmacol. Ther.* **21**, 637–647 (2008). doi:10.1016/j.pupt.2008.02.006 Medline

20. Y. Gu, T. Forostyan, R. Sabbadini, J. Rosenblatt, Epithelial cell extrusion requires the sphingosine-1-phosphate receptor 2 pathway. *J. Cell Biol.* **193**, 667–676 (2011). doi:10.1083/jcb.201010075 Medline

21. M. S. Grace, M. Baxter, E. Dubuis, M. A. Birrell, M. G. Belvisi, Transient receptor potential (TRP) channels in the airway: Role in airway disease. *Br. J. Pharmacol.* **171**, 2593–2607 (2014). doi:10.1111/bph.12538 Medline

22. C. Bae, F. Sachs, P. A. Gottlieb, The mechanosensitive ion channel Piezo1 is inhibited by the peptide GsMTx4. *Biochemistry* **50**, 6295–6300 (2011). doi:10.1021/bi200770q Medline

23. M. M. Price, C. A. Oskeritzian, Y. T. Falanga, K. B. Harikumar, J. C. Allegood, S. E. Alvarez, D. Conrad, J. J. Ryan, S. Milstien, S. Spiegel, A specific sphingosine kinase 1 inhibitor attenuates airway hyperresponsiveness and inflammation in a mast cell–dependent murine model of allergic asthma. *J. Allergy Clin. Immunol.* **131**, 501–511.e1 (2013). doi:10.1016/j.jaci.2012.07.014 Medline

24. C. M. Evans, K. Kim, M. J. Tuvim, B. F. Dickey, Mucus hypersecretion in asthma: Causes and effects. *Curr. Opin. Pulm. Med.* **15**, 4–11 (2009). doi:10.1097/MCP.0b013e32831da8d3 Medline

25. C. M. Evans, D. S. Raclawska, F. Ttofali, D. R. Liptzin, A. A. Fletcher, D. N. Harper, M. A. McGing, M. M. McElwee, O. W. Williams, E. Sanchez, M. G. Roy, K. N. Kindrachuk, T. A. Wynn, H. K. Eltzschig, M. R. Blackburn, M. J. Tuvim, W. J. Janssen, D. A.

Schwartz, B. F. Dickey, The polymeric mucin Muc5ac is required for allergic airway hyperreactivity. *Nat. Commun.* **6**, 6281 (2015). doi:10.1038/ncomms7281 Medline

26. Z. Jia, K. Bao, P. Wei, X. Yu, Y. Zhang, X. Wang, X. Wang, L. Yao, L. Li, P. Wu, W. Yuan, S. Wang, J. Zheng, Y. Hua, M. Hong, EGFR activation-induced decreases in claudin1 promote MUC5AC expression and exacerbate asthma in mice. *Mucosal Immunol.* **14**, 125–134 (2021). doi:10.1038/s41385-020-0272-z Medline

27. S. Piñeiro-Hermida, J. A. Gregory, I. P. López, R. Torrens, C. Ruíz-Martínez, M. Adner, J. G. Pichel, Attenuated airway hyperresponsiveness and mucus secretion in HDM-exposed Igf1r-deficient mice. *Allergy* **72**, 1317–1326 (2017). doi:10.1111/all.13142 Medline

28. A. Hsieh, N. Assadinia, T.-L. Hackett, Airway remodeling heterogeneity in asthma and its relationship to disease outcomes. *Front. Physiol.* **14**, 1113100 (2023). doi:10.3389/fphys.2023.1113100 Medline

29. R. C. Hyzy, W. D. Travis, E. Hanna, S. Lyon-Callo, K. R. Flaherty, K. D. Rosenman, Slow-onset asthma deaths have more eosinophils and health care utilization than rapid-onset deaths. *Respir. Med.* **102**, 1819–1826 (2008). doi:10.1016/j.rmed.2008.06.011 Medline

30. F. ter Woort, J. L. Caswell, L. G. Arroyo, L. Viel, Histologic investigation of airway inflammation in postmortem lung samples from racehorses. *Am. J. Vet. Res.* **79**, 342–347 (2018). doi:10.2460/ajvr.79.3.342 Medline

31. M. Yamaya, Virus infection-induced bronchial asthma exacerbation. *Pulm. Med.* **2012**, 834826 (2012). doi:10.1155/2012/834826 Medline

32. S. T. Holgate, The sentinel role of the airway epithelium in asthma pathogenesis. *Immunol. Rev.* **242**, 205–219 (2011). doi:10.1111/j.1600-065X.2011.01030.x Medline

33. S. T. Holgate, G. Roberts, H. S. Arshad, P. H. Howarth, D. E. Davies, The role of the airway epithelium and its interaction with environmental factors in asthma pathogenesis. *Proc. Am. Thorac. Soc.* **6**, 655–659 (2009). doi:10.1513/pats.200907-072DP Medline

34. A. Tam, S. Wadsworth, D. Dorscheid, S. F. Man, D. D. Sin, The airway epithelium: More than just a structural barrier. *Ther. Adv. Respir. Dis.* **5**, 255–273 (2011). doi:10.1177/1753465810396539 Medline

35. J. A. Mitchel, A. Das, M. J. O'Sullivan, I. T. Stancil, S. J. DeCamp, S. Koehler, O. H. Ocaña, J. P. Butler, J. J. Fredberg, M. A. Nieto, D. Bi, J.-A. Park, In primary airway epithelial cells, the unjamming transition is distinct from the epithelial-to-mesenchymal transition. *Nat. Commun.* **11**, 5053 (2020). doi:10.1038/s41467-020-18841-7 Medline

36. J.-A. Park, J. H. Kim, D. Bi, J. A. Mitchel, N. T. Qazvini, K. Tantisira, C. Y. Park, M. McGill, S.-H. Kim, B. Gweon, J. Notbohm, R. Steward Jr., S. Burger, S. H. Randell, A. T. Kho, D. T. Tambe, C. Hardin, S. A. Shore, E. Israel, D. A. Weitz, D. J. Tschumperlin, E. P. Henske, S. T. Weiss, M. L. Manning, J. P. Butler, J. M. Drazen, J. J. Fredberg, Unjamming and cell shape in the asthmatic airway epithelium. *Nat. Mater.* **14**, 1040–1048 (2015). doi:10.1038/nmat4357 Medline

37. I. T. Stancil, J. E. Michalski, D. Davis-Hall, H. W. Chu, J.-A. Park, C. M. Magin, I. V. Yang, B. J. Smith, E. Dobrinskikh, D. A. Schwartz, Pulmonary fibrosis distal airway epithelia are dynamically and structurally dysfunctional. *Nat. Commun.* **12**, 4566 (2021). doi:10.1038/s41467-021-24853-8 Medline

38. C. E. Deering-Rice, D. Shapiro, E. G. Romero, C. Stockmann, T. S. Bevans, Q. M. Phan, B. L. Stone, B. Fassl, F. Nkoy, D. A. Uchida, R. M. Ward, J. M. Veranth, C. A. Reilly, Activation of Transient Receptor Potential Ankyrin-1 by Insoluble Particulate Material and Association with Asthma. *Am. J. Respir. Cell Mol. Biol.* **53**, 893–901 (2015). doi:10.1165/rcmb.2015-0086OC Medline

39. C. E. Deering-Rice, C. Stockmann, E. G. Romero, Z. Lu, D. Shapiro, B. L. Stone, B. Fassl, F. Nkoy, D. A. Uchida, R. M. Ward, J. M. Veranth, C. A. Reilly, Characterization of Transient Receptor Potential Vanilloid-1 (TRPV1) Variant Activation by Coal Fly Ash Particles and Associations with Altered Transient Receptor Potential Ankyrin-1 (TRPA1) Expression and Asthma. *J. Biol. Chem.* **291**, 24866–24879 (2016). doi:10.1074/jbc.M116.746156 Medline

40. W. C. Wetsel, Sensing hot and cold with TRP channels. *Int. J. Hyperthermia* **27**, 388–398 (2011). doi:10.3109/02656736.2011.554337 Medline

41. N. K. Malavia, C. B. Raub, S. B. Mahon, M. Brenner, R. A. Panettieri Jr., S. C. George, Airway epithelium stimulates smooth muscle proliferation. *Am. J. Respir. Cell Mol. Biol.* **41**, 297–304 (2009). doi:10.1165/rcmb.2008-0358OC Medline

42. E. Fuerst, H. R. Foster, J. P. T. Ward, C. J. Corrigan, D. J. Cousins, G. Woszczek, Sphingosine-1-phosphate induces pro-remodelling response in airway smooth muscle cells. *Allergy* **69**, 1531–1539 (2014). doi:10.1111/all.12489 Medline

43. T. J. A. Maguire, S. Yung, E. Ortiz-Zapater, O. S. Kayode, S. Till, C. Corrigan, L. Q. C. Siew, G. A. Knock, G. Woszczek, Sphingosine-1-phosphate induces airway smooth muscle hyperresponsiveness and proliferation. *J. Allergy Clin. Immunol.* **152**, 1131–1140.e6 (2023). doi:10.1016/j.jaci.2023.05.028 Medline

44. K. M. Akram, L. L. Yates, R. Mongey, S. Rothery, D. C. A. Gaboriau, J. Sanderson, M. Hind, M. Griffiths, C. H. Dean, Live imaging of alveologenesis in precision-cut lung slices reveals dynamic epithelial cell behaviour. *Nat. Commun.* **10**, 1178–1178 (2019). doi:10.1038/s41467-019-09067-3 Medline

45. V. Neuhaus, D. Schaudien, T. Golovina, U.-A. Temann, C. Thompson, T. Lippmann, C. Bersch, O. Pfennig, D. Jonigk, P. Braubach, H.-G. Fieguth, G. Warnecke, V. Yusibov, K. Sewald, A. Braun, Assessment of long-term cultivated human precision-cut lung slices as an ex vivo system for evaluation of chronic cytotoxicity and functionality. *J. Occup. Med. Toxicol.* **12**, 13–13 (2017). doi:10.1186/s12995-017-0158-5 Medline

46. T. Bevans, C. Deering-Rice, C. Stockmann, J. Rower, D. Sakata, C. Reilly, Inhaled Remimazolam Potentiates Inhaled Remifentanil in Rodents. *Anesth. Analg.* **124**, 1484–1490 (2017). doi:10.1213/ANE.0000000000002022 Medline

47. S. A. Glantz, *Primer of Biostatistics* (McGraw-Hill Medical, ed. 7, 2012).
